# Effect of corticospinal and reticulospinal tract damage on spastic muscle tone and mobility: a retrospective observational MRI study

**DOI:** 10.1016/j.ebiom.2025.105824

**Published:** 2025-06-24

**Authors:** Simon Schading-Sassenhausen, Volker Dietz, Patrick Freund

**Affiliations:** a Spinal Cord Injury Center, Balgrist University Hospital, University of Zurich, Forchstrasse 340, 8008, Zurich, Switzerland; b Wellcome Trust Centre for Neuroimaging, UCL Queen Square Institute of Neurology, University College London, London, UK; c Department of Neurophysics, Max Planck Institute for Human Cognitive and Brain Sciences, Leipzig, Germany

**Keywords:** MRI, Spastic muscle tone, Spinal cord injury, Mobility, Functional recovery

## Abstract

**Background:**

Spastic muscle tone, often observed after spinal cord injury (SCI), is thought to contribute to body support during walking at a lower level of neural organisation. Understanding the link between damage to specific spinal tracts and the development of spastic muscle tone and mobility could enhance our knowledge of the neural structures crucial for recovery of function after SCI.

**Methods:**

In this retrospective observational study, MRI-based assessments of descending spinal tract damage were related to the development of spastic muscle tone and mobility. Focal damage to the corticospinal (CST) and reticulospinal tracts (RST) was assessed on transversal T_2_-weighted scans of 49 patients with SCI one month post-injury. The extent of tract damage was then associated with the degree of spastic muscle tone measured by the Modified Ashworth Scale (MAS), and changes in mobility subscore of the Spinal Cord Independence Measure (SCIM).

**Findings:**

The extent of CST damage was predictive for the level of MAS score with a positive relationship (β = 0.043, SE = 0.016, OR: 1.044, 95% CI: 1.014–1.080, *P* = 0.006). This association was negatively modulated by the interaction between CST and RST damage (β = −0.002, SE = 0.001, OR: 0.998, 95% CI: 0.997–0.999, *P* = 0.004)—i.e. extensive RST damage weakened the relationship between CST damage and MAS score. The extent of RST damage was related to the change in SCIM mobility subscore, independent of MAS score (β: −0.683, SE = 0.231, 95% CI: −1.135 to −0.230, *P* = 0.007).

**Interpretation:**

The extent of CST damage, along with the preservation of the RST, reliably predicts spastic muscle tone, while preserved RST structure alone serves as an independent predictor of mobility outcomes. These observations highlight the role of the reticulospinal system in the functional recovery of mobility and may have broader relevance for other neurological conditions with spinal cord involvement.

**Funding:**

10.13039/501100001711Swiss National Science Foundation.

**Research in context**

**Evidence before this study**

We conducted a comprehensive search of relevant articles in PubMed from database inception to September 10, 2024, using the keywords “spinal cord injury”, “spastic muscle tone”, “mobility”, and “MRI” without any restrictions on language or article type. Our search yielded no articles. Thus, it is understudied how specific spinal tract damage and development of spastic muscle tone and, the interconnected mobility after spinal cord injury depend on each other. Hence, our study aims to bridge these critical knowledge gaps.

**Added value of this study**

The study reveals a complex relationship between specific structural spinal tract damage and development of spastic muscle tone and, the interconnected mobility after spinal cord injury. Corticospinal tract damage is associated with increased spastic muscle tone, as measured by the Modified Ashworth Scale. However, this relationship is modulated by reticulospinal tract damage. While extensive corticospinal tract damage alone leads to a stronger spastic muscle tone, the combination with damaged reticulospinal tract structure reduces this relationship. These findings suggest that reticulospinal tract integrity plays a crucial modulatory role in the presence of spastic muscle tone. Preserved reticulospinal tract structure also determines outcome of mobility following spinal cord injury. This differential effect of corticospinal damage but preserved reticulospinal tract structure provides new insights into the neuroanatomical basis of recovery of function on a lower level of neural control after spinal cord injury.

**Implications of all the available evidence**

This study provides novel insights into the neuroanatomical basis of the development of spastic muscle tone and mobility following SCI, revealing a complex interplay between corticospinal and reticulospinal tract damage. The findings have far-reaching implications for the role of brainstem centres in the recovery of function not only after SCI but also for a range of central nervous system disorders such as stroke and multiple sclerosis. By identifying potential early biomarkers for the development of spastic muscle tone and mobility, this research underlines the critical importance of early and ongoing monitoring of tract damage throughout the recovery of function. These discoveries open new avenues for developing targeted interventions and improving prognosis about the outcome of function after SCI.

## Introduction

Spinal cord injury (SCI) is commonly associated, besides sensorimotor deficits and autonomous dysfunction, with the development of spastic muscle tone.^1^^,^^2^ Approximately two-thirds of patients with SCI are anatomically incomplete and will show some degree of motor recovery,^3^ which might be predicted by the emergence of spasticity.^4^ Initially, SCI results in flaccid paresis, inhibiting stepping movements. The development of spastic muscle tone plays a crucial role in regaining mobility in patients with incomplete SCI, effectively compensating for the loss of supraspinal control that causes paresis.^3^^,^^5^^,^^6^ Therefore, spastic muscle tone and mobility are interconnected. The emergence of spastic muscle tone following CNS damage is attributed to an essential part to alterations in mechanical muscle fibre properties, such as the loss of sarcomeres.^7^ This adaptive mechanism responds to the degree of paresis, adjusting to meet the body's functional needs. However, in some cases, particularly in immobilised patients, spasticity can become excessive and thereby reduce quality of life.^8^

Understanding the neural structures involved in the development of altered muscle tone and mobility following SCI is of significant clinical interest. Several clinical and electrophysiological measures are available to provide a prognosis about the outcome of function.^9^^,^^10^ The assessment of specific tract damage might provide an additional accurate prognostic measure for the outcome of mobility and an answer to the question, which neural tracts are essential to allow a recovery of function. The corticospinal and reticulospinal tracts constitute major descending motor pathways with convergent projections onto spinal neurons.^11^, ^12^, ^13^ These tracts are suggested to be differentially involved in the recovery of function^14^ and the emergence of spastic muscle tone^15^ after a damage of the central nervous system. For example, in individuals suffering from a stroke, response amplitudes to a startle stimulus depend on the level of spastic muscle tone.^16^, ^17^, ^18^ Given that the startle stimulus is thought to engage the reticulospinal tract,^19^^,^^20^ it has been proposed that, in conditions involving the loss of corticospinal axons, the reticulospinal pathway might become the dominant route for motor control.^21^, ^22^, ^23^, ^24^ Also, electrophysiological evidence suggests an imbalance between corticospinal and reticulospinal systems influences muscle tone after SCI, with stronger reticulospinal dominance associated with higher spastic muscle tone.^25^

This study aims to directly elucidate the relationship between specific descending tract damage and the development of spastic muscle tone in-vivo, as well as its impact on the recovery of mobility after SCI. We assess how this corticospinal-reticulospinal imbalance is reflected in anatomical tract damage and relates to spastic muscle tone levels and mobility.^25^^,^^26^ We hypothesise that (i) an imbalance between corticospinal tract damage, paired with a dominant reticulospinal system is associated with the level of spastic muscle tone and, that (ii) preserved corticospinal and reticulospinal tract structures are associated with the recovery of mobility. We use axial T_2_-weighted scans to quantify the extent of structural corticospinal and reticulospinal tract damage and explore how the magnitude of individual tract damage leads to both the development of spastic muscle tone and/or recovery of mobility.

## Methods

### Ethics

The clinical data of all participants included in this study was taken from the European Multicentre Study about Spinal Cord Injury (EMSCI) (https://www.emsci.org/). Within the EMSCI-network clinical, functional and electrophysiological data of individuals suffering from a SCI are collected in meanwhile 25 centres according to a standardised protocol. The following request to the data bank was performed: The dataset comprises T_2_-weighted axial scans at ∼30 days post-injury (selected by Dr Dario Pfyffer from individuals who are included in the EMSCI study), identified by PIDs under EMSCI Project SIMON-23 (qry_SIMON_SCIM23SubScores-26-02-2024.xlsx), with corresponding 1- and 12-month SCIM scores and MAS, noting that EMSCI does not assign public accession numbers. Written informed consent was obtained before participation in the study, which was approved by the local Ethics Committee (EK-03/2004). The study was conducted in accordance with the Declaration of Helsinki.

### Study design

This retrospective observational study includes 49 patients with SCI, who underwent routine clinical MR imaging at one month after injury. The patients included were admitted consecutively to rehabilitation in the Spinal Cord Injury Center of the Balgrist University Hospital Zurich, Switzerland between January 2008 and March 2019. Participants with pre-existing neurological or mental disorders, and contraindications to MRI were excluded. Sex was self-reported as part of the standard clinical information system. Race and ethnicity were not collected in the database of this study.

### Clinical measures

The 49 participants underwent standard clinical examination according to the International Standards for Neurological Classification of Spinal Cord Injury (ISNCSCI) at one month after injury (29.8 ± 7.0 days).^27^ Based on this assessment, patients were classified into different categories on the American Spinal Injury Association Impairment Scale (AIS): AIS A (sensory and motor complete), *n* = 7; AIS B (sensory incomplete, motor complete), *n* = 6; AIS C (sensory and motor incomplete), *n* = 7; AIS D (sensory and motor incomplete, able to walk), *n* = 29. The Spinal Cord Independence Measure (SCIM) was conducted at one month and at 12 months after injury to assess the change in patient's mobility using the mobility SCIM subscore.^28^ The level of spastic muscle tone was assessed using the Modified Ashworth Scale (MAS) during routine check-ups after hospital discharge.^29^ The highest MAS score below the injury across all examinations over the observational period of 5 years was taken for this study. Furthermore, the time elapsed between injury and assessment of the measures, as well as the antispastic medication taken prior to the examinations were included in the study.

### Acquisition of MRI images

Conventional clinical sagittal T_1_-weighted (T_1_-w) and T_2_-weighted (T_2_-w) as well as transversal T_2_-w MR images were acquired on 1.5T and 3T clinical scanners at one month after injury. The scanner models included Siemens Avanto (or Avanto^fit^), Siemens Symphony, Siemens Espree, GE Signa HDxt for 1.5T scanners and Siemens Skyra (or Skyra^fit^) and Siemens Verio for 3T scanners. The lesion and focal tract damage were only assessed on the transversal images; however, the sagittal images were used for better orientation and to identify vertebral levels.

### Analysis of MRI images

**Fig. 1:**
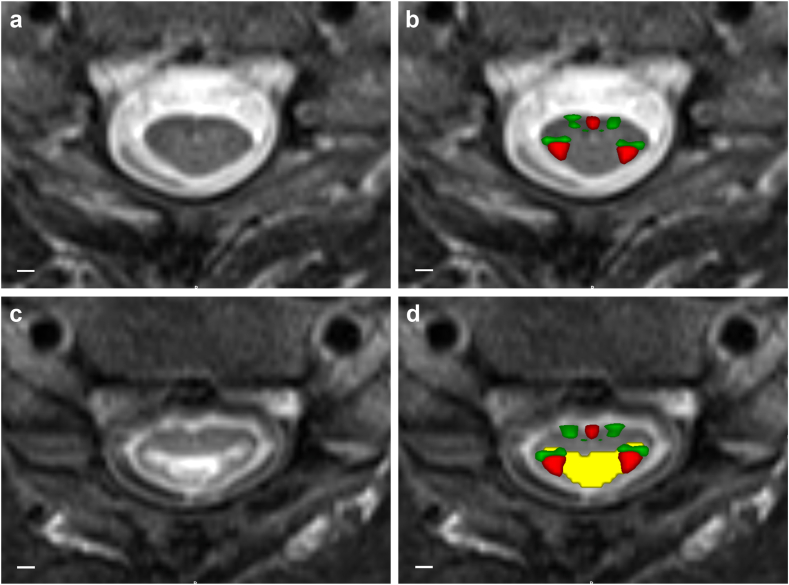
**Assessment of anatomical tract damage**. (**a**) Example of a transversal T_2_-w image of the spinal cord in an uninjured area. (**b**) Overlay of masks of the corticospinal tracts (red) and the reticulospinal tracts (green) on the transversal T_2_-w image. (**c**) Example of a transversal T_2_-w image of an injured spinal cord depicting the hyperintense intramedullary lesion. (**d**) Overlay of masks of the corticospinal tracts (red) and the reticulospinal tracts (green) as well as the lesion segmentation (yellow) on the transversal T_2_-w image. Scale bars, 2 mm.

The extent of focal tissue damage, represented as intramedullary hyperintense area, was assessed on transversal T_2_-w images. This focal lesion was automatically segmented using the deep learning-based algorithm implemented in the *seg_sc_lesion_t2w_sci* task of the *sct_deepseg* algorithm (Spinal Cord Toolbox, version 6.2), which resulted in binary masks of the lesion and the spinal cord.^30^ These binary masks were visually inspected and manually corrected if necessary. Using the spinal cord segmentation, the transversal T_2_-w images were co-registered with the MNI-Poly-AMU template^31^ after manual identification of two vertebral levels—one above and one below the lesion. Next, the spinal cord white matter atlas^32^ was warped into each subject's native space using the transformation fields resulting from co-registration. Based on the warped white matter atlas, binary masks of the corticospinal tract (CST) and the reticulospinal tract (RST) were created. The extent of white matter tract damage was estimated slicewise for both tracts by calculating the percentual overlap between the respective binary masks of the tract and the focal lesion areas. Finally, maximum tract damage was calculated as the largest extent of white matter tract damage across all slices per subject for every tract separately. Fig. 1 shows an example of a transversal T_2_-w image with the corresponding lesion segmentation and both white matter tracts.

### Statistics

All statistical analyses were conducted in RStudio (Version 4.0.5). To examine whether individual tract damage of the CST and RST significantly differs between MAS groups, ANOVA was conducted, which was corrected for the AIS grade, antispastic medication prior to the MAS assessment, and for time interval between the injury and MAS assessment. Post-hoc pairwise comparison of the different MAS groups was conducted using Tukey's correction (*P* < 0.05) to correct for multiple comparison and control for the type I error.

To determine the relationship between damage of the two descending motor tracts and the level of spastic muscle tone, three ordinal logistic regression models (proportional odds model using the clm function in R) were created with the MAS as outcome variable. The predictors of these models were the CST damage (model 1), RST damage (model 2), and the combination of CST and RST tract damage, including the interaction effect (model 3). Moreover, the covariates AIS grade, the time interval between injury and MAS score assessment, as well as antispastic medication were added for correction for these variables. For the calculation of the models' fit, the small-sample corrected Akaike's information criterion (AICc), the Bayesian information criterion (BIC), and the log-likelihood were compared.

Furthermore, the relationship between descending tract damage and change in mobility, reflected in the subscore of the SCIM, was assessed independent of MAS measure. Three linear regression models were created similar to the previous analysis with the predictors CST damage (model 1), RST damage (model 2), and CST and RST tract damage including the interaction effect (model 3). In order to estimate the relationship between tract damage and mobility, the MAS measure and the time elapsed between injury and the assessment were included as covariates. In addition, the models were corrected for antispastic medication. The models' fit was compared using the AICc, BIC, and log-likelihood. It has to be noted that this analysis was conducted only for a subset of patients with SCI (*n* = 33), whose MAS was recorded during the first year after injury together with the follow-up SCIM assessment.

For the evaluation of the individual model coefficients in both the ordinal logistic regression and linear regression models, a significance threshold of *P* < 0.05 was used.

### Role of funders

The funders had no role in the study design, data collection, analysis, interpretation, manuscript preparation, or the decision to submit the manuscript for publication.

## Results

The patients with SCI included in this study were 52.1 ± 17.8 years old (39 male, 10 female). The average time elapsed between injury and MRI examination was 35.8 ± 16.4 days. The clinical examination for MAS was conducted 400.6 ± 427.7 days after injury (range: 29–1805 days). The baseline SCIM measures for the subset of patients (*n* = 33; MAS assessed during the first year after injury), was conducted 29.2 ± 4.5 days after injury and the follow-up measurement 356.4 ± 40.6 days after injury. The level of spastic muscle tone (MAS) amounted to MAS 0: in *n* = 8, MAS 1: in *n* = 9, MAS 2: in *n* = 13, MAS 3: in *n* = 7, and MAS 4: in *n* = 12 patients. Detailed demographics stratified according to sex are summarised in Supplementary Table S1.

**Fig. 2:**
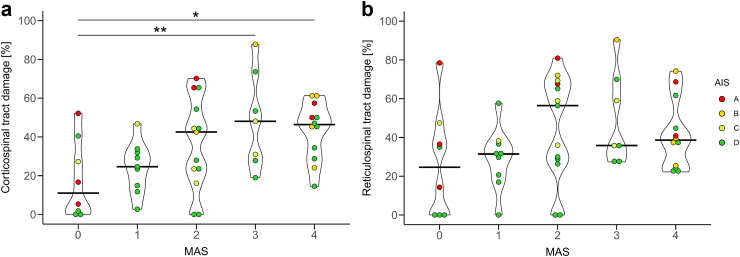
**Tract damage and development of spastic muscle tone (MAS)**. (**a**) Results of the ANOVA comparing corticospinal tract (CST) damage between different MAS. There was a significant main effect of CST damage between groups (*F* (4,44) = 4.07, *P* = 0.007). Significant post-hoc tests between individual MAS groups adjusted for multiple comparison are indicated in the figure. ∗*P* < 0.05, ∗∗*P* < 0.01. (**b**) ANOVA comparing reticulospinal tract (RST) damage between different MAS showing no significant differences between groups. The AIS is colour-coded as indicated in the figure.

First, we assessed whether tract damage differed between MAS grades using ANOVA. The MAS showed a significant main effect for the tract damage of CST (*F* (4,44) = 4.07, *P* = 0.007) but not for the tract damage of RST (*F* (4,44) = 2.11, *P* = 0.10) (Fig. 2). Post-hoc tests revealed that the extent of CST damage affects the MAS level. Patients with a MAS of 3 (difference: 42.0%, 95% CI: 10.1%–74.0%, *P* = 0.005) and those with a MAS of 4 (difference: 31.2%, 95% CI: 1.0%–61.4%, *P* = 0.040) had significantly higher values compared to patients with a MAS of 0.

**Fig. 3:**
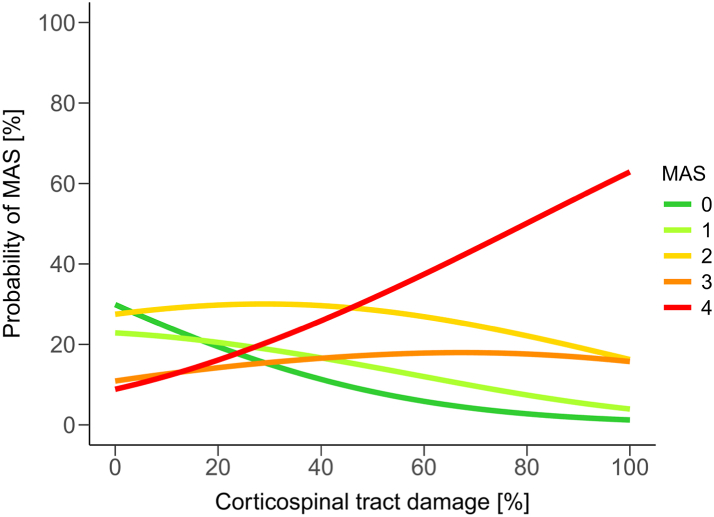
**Effect of corticospinal tract (CST) damage on the development of spastic muscle tone (MAS).** This figure depicts the relationship between CST damage and the level of MAS using an ordinal logistic regression model. The y-axis indicates the probability of a specific level of spastic muscle tone (MAS), while the extent of CST damage is plotted on the x-axis. The different MAS grades are colour-coded as indicated in figure. A significant positive relationship exists between CST damage and MAS grade (OR: 1.044, 95% CI: 1.014–1.080, *P* = 0.006). It suggests that more extensive CST damage is associated with a higher MAS. According to this model, in patients with a CST damage of 0% the predominant MAS grades are 0–2, while the majority of patients with a CST damage of 100% develops severe spastic muscle tone with a MAS of 4.

When assessing the relationship between tract damage and MAS grade using ordinal logistic regression models, CST damage (Model 1) was a significant predictor of MAS grade (β = 0.043, SE = 0.016, Odds Ratio [OR]: 1.044, 95% CI: 1.014–1.080, *P* = 0.006). This positive relationship suggests that greater CST damage is associated with a higher MAS grade, with a 1% increase in tract damage resulting in 4.4% higher odds of an MAS grade increase. Consequently, patients with 100% CST damage predominantly develop a MAS of 4 (62.9%), whereas those with 0% CST damage mainly exhibit MAS grades between 0 and 2 (MAS 0: 30.0%, MAS 1: 22.9%, MAS 2: 27.5%) (Fig. 3). In contrast, RST damage (model 2) was not significantly related to the MAS grade (β = 0.021, SE = 0.014, OR: 1.021, 95% CI: 0.993–1.051, *P* = 0.14). The detailed results of these models are reported in Supplementary Table S2.

**Fig. 4:**
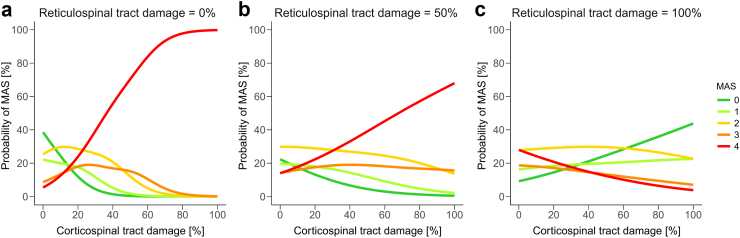
**Interaction of tract damage and development of spastic muscle tone (MAS).** Results of the ordinal logistic regression model similar to Fig. 3. This figure includes the interaction between corticospinal tract (CST) and reticulospinal tract (RST) damage. There was a significant positive relationship between CST damage and MAS, which was negatively modulated by the interaction between CST and RST damage (OR: 0.998, 95% CI: 0.997–0.999, *P* = 0.004). The modulatory effect of RST damage (low–medium–high) in relation to CST damage on MAS grade is depicted. The y-axis indicates the probability of a specific level of spastic muscle tone (MAS), while the extent of CST damage is plotted on the x-axis. The different MAS grades are colour-coded as indicated in the figure. (**a**) Relationship between MAS and CST damage combined with little (0 percent) RST damage. (**b**) Same relationship as in (A) combined with medium (50 percent) RST damage. (**c**) Same relationship as in (A) combined with extensive (100 percent) RST damage. While the majority of patients with extensive CST damage develop severe spasticity (i.e. MAS 4) in presence of little RST damage (panel A), this effect becomes reduced with an increase of RST damage. No more clear tendency in MAS level exists across different ranges of CST damage if RST damage is high (panel C).

The analysis of a modulatory effect due to an interaction between CST and RST damage (Model 3) provided evidence that, in addition to the main effect of CST damage, the interaction between CST and RST damage modulated spastic muscle tone (MAS grade) negatively (β = −0.002, SE = 0.001, OR: 0.998, 95% CI: 0.997–0.999, *P* = 0.004). This suggests that extensive RST damage attenuates the positive effect of CST damage on spastic muscle tone, resulting in a lower MAS grade, with a 0.2% reduction in the odds of an MAS grade increase per unit increase in CST-RST interaction. The resulting distribution of MAS grades is illustrated in Fig. 4 with different degrees of RST damage (low (0%)—medium (50%)—high (100%)) to visualise the modulatory effect of RST damage. With 0% RST damage, the positive association between CST damage and MAS was more pronounced, in the majority of patients with 100% CST damage developing a MAS of 4 (99.8%). Conversely, 0% CST damage predominantly resulted in MAS grades between 0 and 2 (MAS 0: 38.6%, MAS 1: 22.1%, MAS 2: 25.3%). With increasing RST damage, the effect of CST damage on spastic muscle tone was gradually reduced. Thus, in a condition with 100% RST damage, the MAS grade in patients with 100% CST damage resulted in a MAS 0–2 (MAS 0: 43.8%, MAS 1: 22.5%, MAS 2: 22.7%). In contrast, at 0% CST combined with 100% RST damage, no clear tendency for MAS grades was observed (MAS 0: 9.2%, MAS 1: 16.3%, MAS 2: 27.8%, MAS 3: 18.7%, MAS 4: 28.0%).

Table 1 summarises the model fit parameters AICc, BIC, and log-likelihood for all three models to determine which model best describes the data. AICc and BIC were lowest and log-likelihood was highest for the CST and RST interaction model (model 3) suggesting that this model fits the data best.

**Table 1:** Goodness of fit parameters for relationship between tract damage and MAS.

Model	AICc	BIC	Log-likelihood
1: CST	141.5	154.6	−57.8
2: RST	147.8	160.9	−61.0
3: CST ∗ RST	138.9	152.9	−53.1

MAS: Modified Ashworth Scale, CST: corticospinal tracts, RST: reticulospinal tracts, AICc: small-sample corrected Akaike's information criterion, BIC: Bayesian information criterion.

**Fig. 5:**
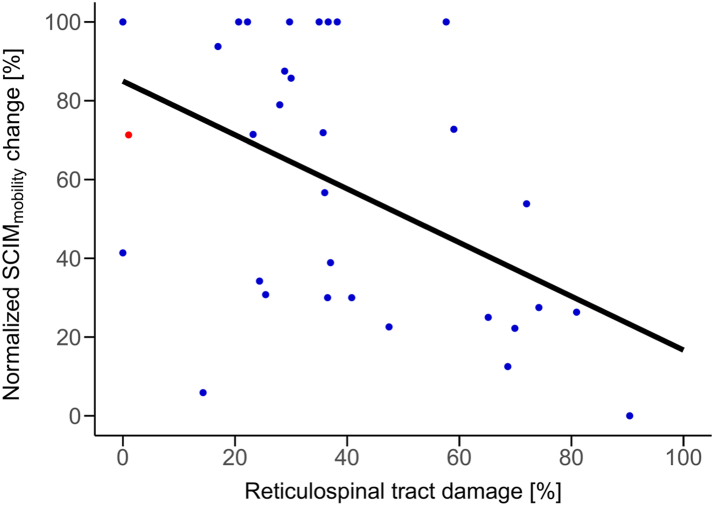
**Effect of tract damage on the change in mobility**. Association between reticulospinal tract (RST) damage and change in mobility subscore of the SCIM as assessed with linear regression. There is a significant negative relationship between RST damage and the change in mobility (β: −0.683, 95% CI: −1.135 to −0.230, *P* = 0.007), i.e. less RST damage is associated with a better recovery in mobility.

The relationship between specific descending tract damage and change in mobility subscore of the SCIM within 12 months from injury was assessed independent of the MAS using linear regression models with MAS as covariate. The extent of preserved RST structure (model 2) was significantly related to the change in mobility (β: −0.683, SE = 0.231, 95% CI: −1.135 to −0.230, *P* = 0.007) (Fig. 5), while the relationship between the extent of preserved CST structure (model 1) and change in mobility was not significant (β: −0.492, SE = 0.281, 95% CI: −1.043 to 0.058, *P* = 0.092). The detailed results of these models are reported in Supplementary Table S3.

Moreover, it was evaluated whether the interaction between CST and RST damage modulates the outcome of recovery in mobility (model 3). No significant interaction effect was found (β: −0.014, SE = 0.008, 95% CI −0.029 to 0.008, *P* = 0.077).

For the calculation of the relationship between preserved motor tract structure and outcome of mobility the model fit of CST damage (model 1), RST damage (model 2), and the interaction of CST and RST damage (model 3) was compared. The goodness of fit parameters are described in Table 2. The data show that the AICc and BIC are lowest, and the log-likelihood is second highest for the RST only model (model 2).

**Table 2:** Goodness of fit parameters for association between tract damage and mobility.

Model	AICc	BIC	Log-likelihood
1: CST	333.6	339.2	−153.9
2: RST	327.5	333.2	−150.8
3: CST ∗ RST	331.3	335.2	−148.4

CST: corticospinal tracts, RST: reticulospinal tracts, AICc: small-sample corrected Akaike's information criterion, BIC: Bayesian information criterion.

## Discussion

This study investigates the relationship between the magnitude of structural corticospinal and reticulospinal tract damage and the development of spastic muscle tone as well as recovery of mobility in patients with anatomically incomplete SCI. We find a nuanced interplay between corticospinal and reticulospinal tract damage in determining the development of spastic muscle tone and recovery of mobility.

Greater corticospinal tract damage, when paired with preserved reticulospinal tract structure, is associated with higher levels of spastic muscle tone. Additionally, the extent of preserved reticulospinal tract emerges as a predictor of mobility outcomes. These observations highlight the critical role of the reticulospinal system in motor recovery after SCI. By elucidating the differential effects of damage to these specific tracts, the study provides new insights into the mechanisms underlying spastic muscle tone and impaired mobility in incomplete SCI. These findings not only enhance our understanding of post-SCI neurological deficit but also allow us to address key questions about the intricate interplay between corticospinal and reticulospinal tract damage and their impact on spastic muscle tone and recovery of mobility.

Spastic muscle tone and mobility are interconnected. According to the observations made here, the extent of CST damage determines the level of spastic muscle tone. This is also supported by a previous study demonstrating indirectly, that the degree of spared cord tissue in the lateral regions was correlated with the magnitude of spasticity.^33^ Crucially, our findings add to this in showing that the level of spastic muscle tone is modulated by the magnitude of preserved RST structure. Spastic muscle tone not only depends on CST damage but also on preserved RST structure, i.e. it is strongest if CST damage is extensive and damage of RST is small. This relationship is in line with the observation made using electrophysiological readouts, which showed that lesser corticospinal but larger reticulospinal drive influences the level of spastic muscle tone in humans suffering a SCI.^25^

According to the results of this study, besides the development of spastic muscle tone, the integrity of descending fibre tracts is also related to the outcome of mobility. While CST function is partially accessible for studies dealing with this question (e.g. transcranial stimulation, somatosensory evoked potentials), this is less feasible for the RST. It is known, for example, that damage of the corticospinal tract after a stroke can strongly affect finger and hand movements. Such a damage can hardly be compensated by other brain areas. Consequently, a quite weak recovery of paralysed fingers usually occurs,^34^^,^^35^ without evidence that intensive training could lead to a better outcome.^36^ In contrast, damage of other brain areas/tracts is followed by a more favourable recovery of proximal limb muscles.^37^, ^38^, ^39^ This might be due to an involvement of motor brainstem centres in the recovery of function. Clearly, preserved RST structure is not only associated with the presence of spastic muscle tone but also with the interconnected outcome of mobility. In a recent publication,^25^ an imbalance between CST and RST tract damage was shown to underlie spastic muscle tone. Our findings suggest that, through its modulatory influence on the CST, the RST helps prevent excessive spastic muscle tone while still permitting some degree of mobility. Unfortunately, no direct method currently exists to isolate and quantify the RST's specific contribution to movement execution in humans. Nevertheless, our observations indicate that better-preserved RST integrity correlates with improved mobility after SCI. The precise extent to which the RST can compensate for CST damage—and thereby facilitate functional recovery—remains to be determined.

There exists preclinical evidence that phylogenetically old motor brainstem centres, specifically the reticulospinal system, play a key role in the automatic coordination of limb muscles not only in animals but also in humans.^40^ Therefore, this system might be involved in the preservation of a basic mobility after stroke or SCI. In fact, indirect evidence exists that the RST is involved in the neural control of automatically performed arm and leg movements in daily life activities: The RST system is suggested to be involved in the neural coupling of hands during bilateral hand movements, such as opening a bottle.^41^ Furthermore, a neural coupling of upper and lower limbs of both sides exists for stepping movements, indicating a brainstem control of locomotion.^42^^,^^43^ The comparison of the model fit indicated that the relationship between preserved RST and mobility is suggestive of a dominant role of RST in the recovery of function following a spinal cord damage. This finding aligns with indirect evidence suggesting that upregulated reticulospinal activity compensates for the loss of corticospinal drive in chronic stroke.^44^

This study has some limitations. The number of patients in this study was relatively low. Thus, statistical power in our analyses was consequentially lower and the absence of a significant effect in some analyses does not rule out a clinically meaningful effect. However, since the data were obtained from routine clinical examinations at an SCI centre, the population should be representative and relevant for clinical practice. Moreover, although we found no evidence that the proportional odds assumption was violated in the ordinal logistic regression models, the use of exact rather than asymptotic methods may be more appropriate given the small sample size. Furthermore, the MAS is a relatively subjective measure and thus prone to inter-examiner variation. However, it represents the universally accepted clinical measure of muscle tone.^29^ Moreover, the focus of interest was to find a general pattern across MAS groups (e.g. comparing severe with mild spasticity), rather than comparing every subgroup to each other. Another critical aspect of using the MAS examination is the variance in the timing of the examinations. To account for this heterogeneity, all statistical analyses were corrected for the time between injury and MAS examination. Furthermore, the focal lesion shows some dynamics during the first weeks after injury, which might affect quantification of the tract damage. However, most changes occur during the first 48 h after injury^45^ and the images in this study were acquired on average 29 days after SCI suggesting that the lesion has stabilised at this time.

In conclusion, this study examined the relevance of descending tract damage for the development of spastic muscle tone and, the interconnected mobility after an SCI. Corticospinal tract damage is associated with increased spastic muscle tone, as measured by the Modified Ashworth Scale. However, this relationship is modulated by the reticulospinal tract, i.e. depends on the amount of preserved reticulospinal tract structure. While extensive corticospinal tract damage alone leads to a stronger spastic muscle tone, the combination of extensive corticospinal tract and preserved reticulospinal tract structure reduces this relationship. These findings suggest that reticulospinal tract integrity plays a crucial modulatory role in the presence of spastic muscle tone and the outcome of mobility following spinal cord injury.

## Contributors

S.S.: Design of study, Analysed the data, wrote the first draft; V.D.: Design of study, Analysed the data, revised the paper; P.F.: Design of study, Analysed the data, revised the paper; S.S. and P.F. accessed and verified the underlying data. All authors read and approved the final version of the manuscript.

## Data sharing statement

The authors certify they have documented all data, methods, materials, and the applied codes used to conduct the research presented. Anonymised data pertaining to the research presented will be available by reasonable request to the corresponding author (patrick.freund@balgrist.ch). For this project, we requested from the database a set of T_2_-weighted axial MRI scans acquired approximately 30 days post-injury. Dr. Dario Pfyffer selected these scans from individuals enrolled in the EMSCI study. The subjects are identified by patient IDs under EMSCI Project SIMON-23 (qry_SIMON_SCIM23SubScores-26-02-2024.xlsx). In addition, corresponding SCIM scores at 1 month and 12 months, as well as MAS values, were included. Note that EMSCI does not assign public accession numbers.

## Declaration of interests

The authors have no competing interest to disclose.
