# Supplementary material for: Effect of corticospinal and reticulospinal tract damage on spastic muscle tone and mobility: a retrospective observational MRI study
Source: eBioMedicine. 2025 Jun 24;118:105824. doi: 10.1016/j.ebiom.2025.105824 (PMC12256215; doi:10.1016/j.ebiom.2025.105824)
Supplement: Supplementary Tables [file mmc1.docx]

**Supplementary Table S1: Demographics stratified according to sex**

|  | **Male (n = 39)** | **Female (n = 10)** |
| --- | --- | --- |
| Age ± SD [years] | 50.3 ± 18.8 | 59.0 ± 11.5 |
| Time MRI after injury ± SD [days] | 34.7 ± 16.0 | 40.0 ± 18.2 |
| Time MAS after injury ± SD [days] | 373.6 ± 420.1 | 505.9 ± 463.4 |
| Time SCIM baseline after injury ± SD [days] | 29.7 ± 4.3 | 26.8 ± 5.4 |
| Time SCIM follow-up after injury ± SD [days] | 361.1 ± 26.3 | 335.3 ± 79.5 |
| AIS [no.] |  |  |
| A | 6 | 1 |
| B | 6 | 0 |
| C | 6 | 1 |
| D | 21 | 8 |
| MAS [no.] |  |  |
| 0 | 6 | 2 |
| 1 | 8 | 1 |
| 2 | 9 | 4 |
| 3 | 5 | 2 |
| 4 | 11 | 1 |

AIS: American Spinal Injury Association Impairment Scale, MAS: Modified Ashworth Scale, SCIM: Spinal Cord Independence Measure

**Supplementary Table S2: MAS ordinal logistic regression models**

| **Model** | **Variable** | **Beta** | **SE** | **OR (95% CI)** | **z-value** | **p-value** |
| --- | --- | --- | --- | --- | --- | --- |
| 1: CST | CST | 0.043 | 0.016 | 1.044 (1.014, 1.080) | 2.746 | 0.006 |
| 2: RST | RST | 0.021 | 0.014 | 1.021 (0.993, 1.051) | 1.471 | 0.14 |
| 3: CST * RST | CST | 0.124 | 0.036 | 1.132 (1.061, 1.225) | 3.435 | 0.0006 |
|  | RST | 0.028 | 0.028 | 1.029 (0.974, 1.090) | 0.999 | 0.32 |
|  | CST:RST | -0.002 | 0.001 | 0.998 (0.997, 0.999) | -2.864 | 0.004 |

**Supplementary Table S3: SCIM Linear regression models**

| **Model** | **Variable** | **Beta (95% CI)** | **SE** | **t-value** | **p-value** |
| --- | --- | --- | --- | --- | --- |
| 1: CST | CST | -0.492 (-1.043, 0.058) | 0.281 | -1.752 | 0.092 |
| 2: RST | RST | -0.683 (-1.135, -0.230) | 0.231 | -2.958 | 0.007 |
| 3: CST * RST | CST | 0.838 (-0.169, 1.844) | 0.513 | 1.631 | 0.12 |
|  | RST | -0.332 (-1.182, 0.519) | 0.434 | -0.764 | 0.45 |
|  | CST:RST | -0.014 (-0.029, 0.001) | 0.008 | -1.854 | 0.077 |
